# Asian Zika Virus Isolate Significantly Changes the Transcriptional Profile and Alternative RNA Splicing Events in a Neuroblastoma Cell Line

**DOI:** 10.3390/v12050510

**Published:** 2020-05-05

**Authors:** Gaston Bonenfant, Ryan Meng, Carl Shotwell, Pheonah Badu, Anne F. Payne, Alexander T. Ciota, Morgan A. Sammons, J. Andrew Berglund, Cara T. Pager

**Affiliations:** 1 Department of Biological Sciences, University at Albany-SUNY, Albany, NY 12222, USA; gbonenfant@albany.edu (G.B.); pbadu@albany.edu (P.B.); masammons@albany.edu (M.A.S.); aberglund@albany.edu (J.A.B.); 2 The RNA Institute, University at Albany-SUNY, Albany, NY 12222, USA; rmeng@albany.edu (R.M.); cshotwell@albany.edu (C.S.); 3 Department of Biochemistry and Molecular Biology, College of Medicine, University of Florida, Gainesville, FL 32610, USA; 4 Wadsworth Center, New York State Department of Health (NYSDOH), Slingerlands, NY 12159, USA; anne.payne@health.ny.gov (A.F.P.); alexander.ciota@health.ny.gov (A.T.C.); 5 Department of Biomedical Sciences, University at Albany-SUNY, School of Public Health, Rensselaer, NY 12144, USA

**Keywords:** Zika virus, Flavivirus, SH-SY5Y, transcriptome, alternative splicing

## Abstract

The alternative splicing of pre-mRNAs expands a single genetic blueprint to encode multiple, functionally diverse protein isoforms. Viruses have previously been shown to interact with, depend on, and alter host splicing machinery. The consequences, however, incited by viral infection on the global alternative slicing (AS) landscape are under-appreciated. Here, we investigated the transcriptional and alternative splicing profile of neuronal cells infected with a contemporary Puerto Rican Zika virus (ZIKV^PR^) isolate, an isolate of the prototypical Ugandan ZIKV (ZIKV^MR^), and dengue virus 2 (DENV2). Our analyses revealed that ZIKV^PR^ induced significantly more differential changes in expressed genes compared to ZIKV^MR^ or DENV2, despite all three viruses showing equivalent infectivity and viral RNA levels. Consistent with the transcriptional profile, ZIKV^PR^ induced a higher number of alternative splicing events compared to ZIKV^MR^ or DENV2, and gene ontology analyses highlighted alternative splicing changes in genes associated with mRNA splicing. In summary, we show that ZIKV affects cellular RNA homeostasis not only at the transcriptional levels but also through the alternative splicing of cellular transcripts. These findings could provide new molecular insights into the neuropathologies associated with this virus.

## 1. Introduction

Zika virus (ZIKV) is a re-emerging mosquito-borne flavivirus that is classified within the *Flaviviridae* family. Other notable flaviviruses include dengue virus (DENV), yellow fever virus (YFV), West Nile virus (WNV), and tick-borne encephalitis virus (TBEV), all of which are primarily transmitted via the bite of an infected mosquito or tick [1]. Flavivirus infections rarely result in death, and common symptoms include a maculopapular rash, a fever, and achy joints [2]. ZIKV was first identified in 1947 in the Zika forest in Uganda [3,4]. Until the early 2000s, only thirteen confirmed ZIKV infections in humans were reported [5,6,7,8]. The first major outbreak of ZIKV occurred in 2007 on Yap Island [9], followed by a 2010 outbreak in Cambodia [10], and an outbreak in French Polynesia in 2013 that resulted in more than 29,000 human infections [11]. This Asian lineage of ZIKV expanded west, and in 2015, efforts were redirected towards understanding the link between ZIKV infection and the associated neurological pathologies that are now termed Congenital Zika Syndrome (CZS) [12,13]. To date, there are no antivirals or a licensed vaccine to prevent ZIKV infection. Therefore, to develop effective therapies and thus limit the symptoms associated with ZIKV infection, it is critical to understand virus–host interactions and ZIKV pathogenesis.

The striking feature of the 2015 ZIKV outbreak in the Americas was the correlation between prenatal ZIKV infection and devastating consequences for fetal brain development—resulting in microcephaly, cortical malformations, and intracranial calcifications [14,15,16,17]—and the increased number of cases of Guillain–Barré syndrome in adults [18,19,20,21]. As a first step to elucidating ZIKV-directed mechanisms resulting in neurological anomalies, studies using in vitro, ex vivo, primary cell, and in vivo mouse infection models were undertaken. These studies determined that ZIKV infected neuroepithelial stem cells and radial glia cells, resulted in cell cycle arrest, altered differentiation, increased cell death, and altered thicknesses of neuronal layers [22,23,24,25,26]. These outcomes at the cellular level were the result of ZIKV disrupting centrosomes, changing the cell division plane, inducing apoptosis, and altering signaling pathways [14,22,23,24,25,27,28,29]. At the genetic level, ZIKV was shown to dysregulate the transcription of cell-cycle, DNA repair, immune response, cell death, and microcephaly genes [14,22,23,24,25,27,28,29]. Interestingly, differences in the infectivity toward neural stem cells and other neuronal cell lines of the original ZIKV strain isolated in Uganda in 1947 and Asian lineage isolates, including those isolated from the 2015 outbreak in the Americas, have been reported [27,29,30,31,32,33,34,35]. Despite these reported infectivity differences, RNA-seq studies showed that the changes in the transcriptome were less dramatic [14], suggesting that changes in gene expression alone do not entirely explain ZIKV neuropathologies.

We recently showed that during ZIKV infection, HuR (or ELAVL1) is re-localized from the nucleus to ZIKV replication sites [36]. Since the ELAVL family of proteins regulate mRNA splicing and stability [37,38], we posited that the re-localization of certain RNA-binding proteins, such as HuR, could impact RNA transcription as well as mRNA splicing and stability and thus contribute to the dysregulation of cellular pathways critical for neuronal development. Indeed, molecular diversity within the central nervous system is in part the result of alternative splicing events [39]. Studies of developing cortices in primates [40] and rodents [41] showed variation in alternative exons, and brain- or neuron-specific splicing patterns changed dramatically during development [42,43]. Moreover, the temporal and cell-type specific regulation of alternative splicing (AS) events was largely due to the recognition of regulatory sequences within pre-mRNA transcripts by RNA-binding proteins (RBPs) enriched in neurons, such as Rbfox and neuronal ELAVL proteins [39].

In this study, we used RNA-seq to investigate the transcriptional profiles and alternative splicing events in a neuroblastoma cell line following infection with a modern isolate of ZIKV circulating in the Americas (PRVABC59; ZIKV^PR^), the original 1947 ZIKV isolate from Uganda (MR766; ZIKV^MR^), and DENV2, isolated in Peru in 1996. The analysis of global transcription revealed seven times more changes in gene expression following infection with ZIKV^PR^ compared to that following infection with ZIKV^MR^ or DENV2. Moreover, the number of virus-induced alternative splicing events correlated with the transcriptional profile of each virus infection, where infection with ZIKV^PR^ resulted in many more alternative splicing events than ZIKV^MR^ or DENV2 infection. Our study highlights an overlooked impact caused by viral infection on the host and establishes the foundation to further investigate the impact of specific alternatively spliced genes on neuronal development and the subsequent neuropathologies observed following ZIKV infection.

## 2. Materials and Methods

### 2.1. Cell Maintenance

SH-SY5Y neuroblastoma cells (ATCC CRL-2266) were cultured at 37 °C with 5% CO_2_ and maintained in Eagle’s minimum essential medium (Sigma, St. Louis, MO, USA), F-12 Ham with NaHCO_3_ (Sigma), and 10% fetal bovine serum (FBS; Seradigm). *Aedes albopictus* cells (C6/36; ATCC CRL-1660) were grown at 27 °C with 5% CO_2_ and maintained in Eagle’s minimum essential medium supplemented with 10% FBS, sodium pyruvate (0.055 g/L; Life Technologies, Carlsbad, CA, USA), Fungizone (125 μg/L; Life Technologies), and penicillin and streptomycin (50,000 units/L penicillin, 0.05 g/L streptomycin; Life Technologies).

### 2.2. Preparation of Virus Stocks

ZIKV^MR^ (Uganda MR766 strain) was a gift from Dr. Brett Lindenbach (Yale University), and ZIKV^PR^ (Puerto Rico PRVABC59 strain) was a gift from Dr. Laura Kramer (Wadsworth Center NYDOH) and the CDC. Aliquots of DENV2 (OBS-2629) isolated in Peru in 1996 were gifted to the Pager laboratory by Dr. Alexander Ciota (Wadsworth Center NYDOH). To create virus stocks, C6/36 cells were seeded into 15 cm tissue culture plates or T75 flasks, and the cells were infected near confluence. In particular, a viral master mix was prepared (5 mL of PBS and 500 μL of virus aliquot per plate), the medium was aspirated from each plate, and the viral master mix was added to cells. The plate was returned to the incubator for one hour with rocking every 15 min. After the one-hour incubation, 10 mL of complete medium was added, and the plate was returned to the 27 °C incubator for seven days. After seven days, medium was transferred to 50 mL conical tubes, centrifuged at 1000× *g* for 5 min, and 500 μL aliquots of the viruses were prepared and stored at −80 °C. To ensure successful infection, 3 mL of TRIzol reagent was added to the culture plate and RNA was extracted for RT-qPCR analysis. Virus titers were determined by plaque assays as previously described [27]. 

### 2.3. Viral Infections

SH-SY5Y cells were seeded in 6 cm tissue culture plates with 3 mL of complete medium. When cells neared 90% confluence, a control plate of cells was counted to determine the multiplicity of infection (moi). SH-SY5Y cells were infected at a moi of 5. Specifically, the appropriate amount of virus was diluted in PBS to a 1.5 mL total volume, which was then added to each plate. The plates were returned to the 37 °C incubator for one hour with rocking every 15 min, after which 1.5 mL of complete medium was added. The cells were harvested at one day post-infection.

### 2.4. Harvest of Virus-Infected Cells

Cells were harvested by first aspirating the medium from the tissue culture plates, gently washing the cells with 1 mL of PBS, and lysing the cells in 500 μL of TRIzol reagent (Life Technologies). The cells in TRIzol were manually scraped and transferred to a 1.5 mL tube, and RNA was extracted following the manufacturers’ recommendations. Following ethanol cleanup, RNA was resuspended in 20 μL of Ambion 0.2 μm filtered water.

### 2.5. Plaque Assays

Viral titers, as determined by plaque assays, for ZIKV were carried out as has previously been described [36]. Briefly, a monolayer of Vero cells on six-well plates were infected with serial dilutions of virus prepared in PBS. After a one-hour incubation, a 1:1 overlay (1.2% oxoid agar and modified DMEM) was added to each well. After solidifying at RT, the plates were returned to the incubator for 4 days. Plaques were developed using 1% crystal violet in 20% methanol. Plaque assays for DENV were performed with slight modifications. The 1:1 overlay mixture was composed of 2× MEM, 10% FBS and 1.2% oxoid agar. Additionally, plaques were developed using neutral red staining. One day prior to plaque visualization, an additional oxoid agar overlay was prepared by adding 2 mL of neutral red stock (0.33%) per 100 mL of overlay. The agar was melted, and 3 mL were added to each well. The plate was left at RT for 10 min for the agar to solidify and then returned to the incubator overnight for the stain to diffuse into the cells of the monolayer.

### 2.6. Immunofluorescence Analysis

SH-SY5Y cells were seeded into 24-well plates treated with poly-d-lysine (1 mg/mL; Sigma). When cells reached 90% confluence, 2 wells were trypsinized and counted to determine the moi. Similar to infection in the 6 cm tissue culture plates, the SH-SY5Y cells were infected at a moi of 5 in a total viral master mix volume of 300 μL. One hour after the addition of virus, 600 μL of complete medium was added to each well. One day post-infection, the cells were washed twice with PBS, fixed with 4% paraformaldehyde in PBS for 10 min at room temperature, and then permeabilized with 100% iced methanol for 10 min. The cells were washed at room temperature in blocking buffer (PBS-1% fish gelatin (FG); Sigma) three times for 15 min. Mouse-anti-J2 dsRNA antibody (Scicons) was diluted in blocking buffer (1:250 *v*/*v*), added to the appropriate wells, and incubated overnight at 4 °C. Donkey-anti-mouse IgG Alexa Fluor-488 antibody (Invitrogen) diluted in blocking buffer (1:200 *v*/*v*) was added for 1 h at room temperature in the dark. Hoechst-33342 (Life Technologies) was applied for 15 min. Before and after the application of antibodies, the cells were washed with blocking buffer three times for 15 min. Finally, the cells were washed twice with PBS for 5 min and visualized on the Evos FL cell imaging system (ThermoFisher Scientific).

### 2.7. RT-qPCR

To confirm ZIKV and DENV infection prior to the preparation of RNA-seq libraries, RT-qPCR was performed with virus-specific primers (Table S1), and the amount of viral RNA was determined relative to β-actin mRNA levels. RNA for each sample (100 ng) and 10 μM forward and reverse primers were added to the RT-qPCR master mix containing RT enhancer, Hot-Start master mix, and Verso Enzyme mix (Life Technologies). Reactions were carried out beginning with reverse transcription at 50 °C for 15 min. The RT enzyme was heat-inactivated at 95 °C for 5 min, followed by 35 cycles of 20 s denaturation at 95 °C, annealing at 60 °C for 30 s, and extension at 72 °C for 1 min.

### 2.8. Preparation of Libraries for RNA-Seq

RNA-seq analysis libraries were prepared using the NEBNext^®^ Poly(A) Magnetic Isolation Module (NEB E7490), NEBNext^®^ Ultra™ Directional RNA Library Prep Kit for Illumina^®^ (NEB E7760), and NEBNext^®^ Multiplex Oligos for Illumina^®^ (NEB E7335S). Three libraries were concurrently prepared from three independent experiments of mock-, ZIKV- (MR766 and PRVABC59), and DENV2-infected SH-SY5Y cells. The poly(A) mRNA was isolated via magnetic beads (NEB #E7490) from the TRIzol-extracted RNA. In brief, RNA (100 ng) was diluted in nuclease-free water to a final volume of 50 μL, and oligo dT beads in 50 μL of RNA binding buffer were added. Following a 65 °C incubation for 5 min, samples were cooled to 4 °C, left at room temperature for 5 min, and washed. Next, 50 μL of Tris buffer was added to each sample, mixed, and incubated at 80 °C for 2 min. After cooling to room temperature, 50 μL of RNA binding buffer was added and incubated for 5 min. Beads were washed, the supernatant was aspirated, and mRNA was eluted by the addition of 11.5 μL of First Strand Synthesis Reaction Buffer and Random Primer Mix. Prior to the synthesis of cDNA, samples were placed in a thermal cycler set to 94 °C for 15 min to fragment the mRNA, which was then transferred to a new tube and placed on ice.

First strand cDNA synthesis reactions were assembled according to the manufacturers’ specifications and added to samples, which were placed in a thermal cycler set to 25°C for 10 min, 42 °C for 15 min, 70 °C for 15 min, and a 4 °C hold. Second strand synthesis was assembled as instructed by the manufacturer, and samples were incubated for one hour at 65 °C. Double-stranded cDNA was purified using SPRIselect Beads. Briefly, cDNA was incubated with beads at room temperature for 5 min, the supernatant was aspirated, and the beads were washed three times with freshly prepared 80% ethanol. After the final wash, beads were air-dried and DNA was eluted by adding 53 μL of 0.1× TE buffer, vortexing, and incubating at room temperature for 2 min, and then the supernatant was transferred to a new tube.

Adaptor ligation was performed using NEBNext Ultra II End Prep Reaction according to NEB’s instructions. NEBNext adaptors diluted 5-fold in iced adaptor dilution buffer, ligation enhancer, and ligation master mix were added to each tube, gently mixed, and incubated at 20 °C for 15 min. Following the 15-min incubation, the USER Enzyme was added to each reaction, which was incubated at 37 °C for 15 min. The ligation reactions were purified using SPRIselect beads as described above, though DNA was eluted in 17 μL of 0.1× TE buffer. The eluted DNA was transferred to a new tube, and PCR enrichment of the adaptor ligated DNA was performed using NEBNext Multiplex Oligos for Illumina (Set 1, NEB #E7335). The PCR reaction mix was assembled according to the manufacturers’ recommendations and placed in a thermal cycler set to the recommended conditions. Based on the amount of input material, 10 cycles were carried out. PCR reactions were purified using SPRIselect beads, eluted in 23 μL of 0.1× TE buffer, and transferred to clean tubes. Prior to sequencing, libraries were checked for purity via a bioanalyzer and RT-PCR. RNA-seq was performed using the Illumina NextSeq500.

### 2.9. Bioinformatics Preprocessing

Raw sequencing reads were analyzed for quality using FASTQC [44]. Following quality and control analysis, raw sequencing reads were aligned back to the hg19 reference genome using STAR [45] for AS analysis. Post alignment, BAM files were indexed using Samtools [46]. Count tables were generated with Salmon [47] for differential gene expression (DGE) analysis. 

### 2.10. Differential Gene Expression (DGE) Analysis and Alternative Splicing

DGE analysis was performed using DESeq2 [38]. Genes that exhibited an adjusted P value of less than or equal to 0.05 were deemed statistically significant. Gene enrichment analysis of the statistically significant differential gene expression was done with Panther [48]. Heatmaps and Volcano plots were produced using the Enhanced Volcano [49] and Pheatmap [50] packages, respectively, in R [51].

Differences in splicing between the individual libraries were assessed using replicate multivariate analysis of transcript splicing (rMATS) [52]. Splicing events were filtered using a custom Python [53] script utilizing a cutoff of an absolute value of change in Percent Spliced-In (PSI) of greater than or equal to 0.10 and a false-discovery rate (FDR) of less than or equal to 0.05. Individual events were visualized using Sashimi plots [54] utilizing the rmats2sashimiplot tool (http://www.mimg.ucla.edu/faculty/xing/rmats2sashimiplot/). 

### 2.11. Alternative Splicing Analyses

Following cell infection and TRIzol extraction, RNA concentrations were determined using a Nanodrop, and 500 ng of each sample was used in a reverse transcription with SuperScript IV (Life Technologies) using random hexamer primers (IDT). Half of the suggested amount of Superscript was used in these reactions. The cDNA then underwent PCR for 30 cycles using NEB’s 2× Taq Master Mix and the primer sets listed in Table S1, which were designed for each gene within the exons flanking the included/excluded exon. The resulting products were then run by Fragment Analyzer capillary electrophoresis using the DNF905 1-500 base pair kit (Agilent). Quantification was performed using the following equation: Percent spliced in (PSI) = ((RFU of Inclusion Band)/(RFU Inclusion Band+RFU Exclusion Band))×100%, where RFU represents Relative Fluorescence Units. Graphs were made using the GraphPad Prism software, and unpaired t tests were performed in the program to determine significance.

### 2.12. Statistics

For Figure S2a–e, reads were aligned to the hg38 genome using HISAT2, followed by the quantification of features using FeatureCounts. Transcript counts were used for analysis via Limma-voom with no gene annotation file. Lowly expressed genes were filtered out based on counts per million (cpm) values < 1.0 or samples containing 0 genes. Genes were filtered based on a minimum log2 fold change of 0.5 with a *p*-value adjusted threshold [55] of 0.05. Finally, genes were normalized using the TMM method. The job-dependencies used and versions include Bioconductor-limma (Version 3.34.9), bioconducter-edge (Version 3.20.7), and r-statmod (Version 1.4.30).

### 2.13. Data Access

The sequencing data from RNA-Seq were deposited in the NCBI GEO and are available under accession number GEO: GSE149775.

## 3. Results

### 3.1. SH-SY5Y Cells are Permissive for Both Zika Virus Isolates and Dengue Virus

Phylogenetic analyses have revealed a divergence of African Zika virus (MR766) into a distinct Asian ZIKV lineage [56,57]. Notably, the neurological disorders, such as congenital Zika syndrome in newborns and Guillain–Barré syndrome in adults, have been causally linked to the Asian lineage ZIKV strain [58]. While changes in the transcriptional landscape have been linked to the neuropathologies associated with ZIKV infection, the effect of ZIKV on other cellular mRNA pathways is understudied. Our goal in this study was to investigate the consequence of ZIKV infection for alternative splicing. To this end, we surveyed the transcriptional and alternative splicing (AS) landscape induced by ZIKV infection in SH-SY5Y, a neuroblastoma cell line. Because we were interested in deciphering putative molecular changes contributing to the developmental and neurological anomalies associated with the 2015 ZIKV outbreak, we compared the differences in gene expression and AS resulting from infection with a modern ZIKV strain isolated in Puerto Rico in 2015 (PRVABC59; ZIKV^PR^) [59] to those resulting from that with the original ZIKV isolate from Uganda (MR766; ZIKV^MR^) [3] as well as to those resulting from that with a flavivirus that is not known to cause neuropathies, namely, a serotype 2 dengue virus isolate from Peru (DENV2). 

**Figure 1:**
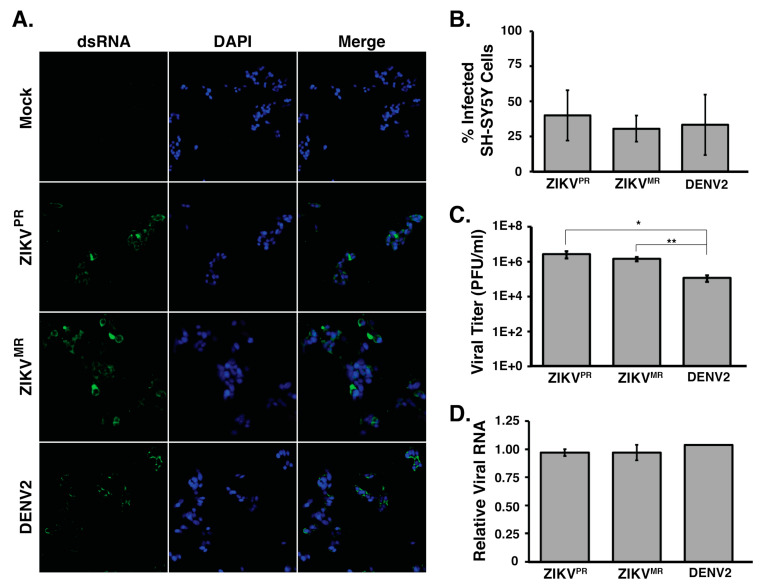
SH-SY5Y cells are infected by ZIKV and DENV2. (**A**) Immunofluorescence images of virus-infected SH-SY5Y cells. SH-SY5Y cells seeded in 48-well plates were infected at a moi of 5 and fixed 24 h post infection. Virus-infected cells were visualized using an antibody that detects the replication intermediate dsRNA, and all cells in the field of view were visualized by staining cell nuclei with Hoechst and at 10× magnification. (**B**) Quantification of the percentage of infected cells. Uninfected and virus-infected SH-SY5Y cells seeded in 48-well plates were fixed and stained for dsRNA and Hoechst. A 2F0× objective was used to image three sections per well per virus where more than 400 cells for each independent infection were counted. The percentages of infected cells were determined. At least three biological replicates were performed. (**C**) Titers of virus released into the medium from ZIKV and DENV2-infected SH-SY5Y cells were determined by plaque assays. (**D**) RT-qPCR analysis of SH-SY5Y cells infected at a moi of 5. Primers targeting the coding regions of each virus were used along with primers for β-actin mRNA. Relative viral RNA levels were calculated by standardizing relative fluorescent units at Ct for each virus against β-actin mRNA. Error bars represent standard deviations established from three independent infections. No significant difference was determined for the number of cells counted in panel 1B, or the relative abundance of viral RNA in panel 1D. For statistical analysis, two-tailed student T-tests were performed (* *p* < 0.05; ** *p* < 0.01).

We first examined the permissiveness of SH-SY5Y cells to ZIKV^PR^, ZIKV^MR^, and DENV2 infection. In particular, SH-SY5Y cells were infected at a moi of 5, and at 24 h post infection, we examined virus infection (Figure 1). Specifically, we determined the extent of infection by fixing and processing cells for immunofluorescence analysis. Using an antibody that specifically detected double-stranded RNA, an intermediate of flavivirus replication (Figure 1a), we observed that 31% to 40% of all cells were infected with ZIKV^PR^, ZIKV^MR^, and DENV2 (Figure 1b). Analysis of the infectious particles released into the culture medium by plaque assays showed that ZIKV^PR^ and ZIKV^MR^ produced similar viral titers in SH-SY5Y cells. Despite DENV2 showing similar levels of virus infection in SH-SY5Y cells, the number of infectious DENV2 particles released into the medium was lower compared to that from ZIKV-infections (Figure 1c). We also isolated total RNA and determined the relative abundance of viral RNA by RT-qPCR. To this end, we used primers specific to each virus and normalized the Ct values to those obtained for β-actin mRNA in each sample. RT-qPCR revealed no statistically significant difference in the viral RNA abundance between each virus (Figure 1d). Together, these data indicate that ZIKV^PR^, ZIKV^MR^, and DENV2 show similar infection levels in SH-SY5Y cells. Moreover, these data demonstrate that SH-SY5Y cells are equally permissive for the different flavivirus infections.

### 3.2. ZIKV^PR^ Significantly Alters Differential Gene Expression Compared to ZIKV^MR^ and DENV2

**Figure 2:**
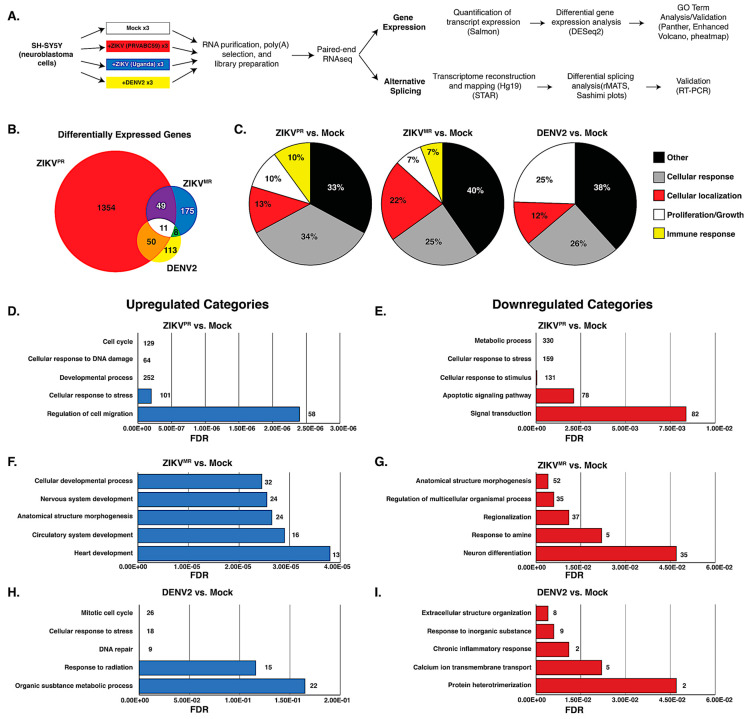
Transcriptome analysis of mock- and virus-infected SH-SY5Y cells. (**A**) Schematic showing the pipeline from cells to differential gene expression and alternative splicing analysis. (**B**) Venn diagram of differentially expressed (DE) transcripts between cells infected with different viruses versus mock-infected SH-SY5Y cells. DE transcripts from ZIKV^PR^ versus mock, ZIKV^MR^ versus mock, and DENV2 versus mock are highlighted in the red, yellow, and blue circles, respectively. (**C**) All differentially expressed genes for each condition were input into ShinyGO(2.0), and the top 25 biological GO terms were categorized into five types, with the distribution of each type presented in a pie chart. (**D**,**F**,**H**) Top five functional categories derived from statistically significant upregulated genes for the indicated condition. (**E**,**G**,**I**) Top five functional categories derived from statistically significant downregulated genes for the indicated condition. GO terms are annotated on the y-axes, and false discovery rates (FDR) are represented on the x-axes.

To compare changes in the transcriptome, we examined poly(A)-selected transcripts isolated from mock- or virus-infected SH-SY5Y cells 24 h post infection and performed RNA-seq analysis on the Illumina platform (Figure 2a). Prior to library preparation, we confirmed virus infection by RT-qPCR (Figure 1c). The analyses were derived from 75-nt paired-end reads from three biological replicates. We obtained a total of 35–48 million reads from the three independent experiments that were mapped back to the reference human genome. Transcript expression was quantified using Salmon [47], and differential expression (DE) analysis was performed using DESeq2 [60] (Figure 2a). We observed a significant difference in DE resulting from ZIKV^PR^ infection, demonstrating that the modern isolate of ZIKV dramatically changed the transcriptome compared to infections with the African isolate ZIKV^MR^ and DENV2 (Figure 2b). A total of 1464 genes were differentially expressed in cells infected with ZIKV^PR^, nearly 6- and 8-fold higher compared to ZIKV^MR^- and DENV2-induced DE genes, respectively (Figure 2b). Of the ZIKV^PR^-induced DE genes, 703 and 651 were upregulated and downregulated, respectively (Figure S1). Eleven DE genes were common to the three flaviviruses, while 50 DE genes were shared between ZIKV^PR^ and DENV2, and 49 DE genes were common between the two ZIKV isolates (Figure 2b).

To determine the gene categories broadly affected by these viruses, gene ontology (GO)-term analysis was performed on all statistically significant DE genes for infection with each virus compared to mock. The top 25 GO-terms were divided into five different categories (cellular response, cellular localization, cell proliferation/growth, immune response, and other) and plotted as pie graphs (Figure 2c). Interestingly, ZIKV^PR^ induced a greater percentage of genes related to the cellular response compared to ZIKV^MR^ and DENV (Figure 2c), while ZIKV^MR^ affected cellular genes influencing cellular localization (Figure 2c). Additionally, DENV2-altered genes were more heavily linked to cell proliferation and growth compared to those altered by either ZIKV strain. Notably, only ZIKV-infected cells had GO terms associated with the immune response (Figure 2c). In SH-SY5Y cells infected with ZIKV^MR^, 6% of DE genes were categorized under immune response, while 10% of the genes DE in cells infected with ZIKV^PR^ were immune response genes (Figure 2c). 

We also examined the top 10 DE genes from each infection. Notably, the top DE genes in ZIKV^PR^ infection were all upregulated, while in ZIKV^MR^- and DENV2-infected cells, the top 10 DE genes were upregulated and downregulated (Figure S2). Moreover, we observed little-to-no overlap among the top 10 DE genes between the viruses (Figure S2). Indeed, only HSPA5 abundance was dramatically and modestly upregulated in ZIKV^PR^- and ZIKV^MR^-infected cells, respectively. Three (ATF3, DDIT3, and HSPA5) of the top 10 genes upregulated in ZIKV^PR^ are involved in the stress response, and two genes were associated with lipid metabolism (LDLR and SREBF1). Interestingly, three of the top ten upregulated genes in ZIKV^PR^ were non-coding RNAs (SNHG15, SNHG17, and OLMALINC) (Figure S2). GO-term analysis outside the top 10 revealed a large number of genes related to ER stress, protein misfolding, and PERK-mediated apoptosis.

To examine possible links among the top 10 DE genes between each set of virus- and mock-infected cells, the top 10 DE genes were submitted to STRING, an online platform that is part of the ELIXIR infrastructure used to compare functional associations between proteins [61]. Connections between different proteins were only found when comparing ZIKV^PR^- to mock-infected cells (Supplemental Figure S2f), and these connected nodes were involved in PERK-mediated apoptosis (Supplemental Figure S2f). For example, apoptotic activation by PERK occurs when the ATF4/ATF3 complex activates DDIT3, which in turn activates GADD34 [62]. These data suggest that ZIKV^PR^ impacts the expression of genes associated with ER stress at multiple nodes.

We next performed GO-term analysis on genes that were differentially upregulated or downregulated during virus infection. Consistent with earlier transcriptomic studies, ZIKV^PR^ modulated the levels of genes associated with the cell cycle, the cellular response to DNA damage and stress, and apoptosis [22,23,24,25,63] (Figure 2d,e). Indeed, the “cellular response to stress” was overrepresented in both upregulated and downregulated genes for ZIKV^PR^-infected cells (Figure 2d–e). Of the top five upregulated GO terms, only ZIKV-infected cells had terms linked to developmental processes (Figure 2d–g), while those genes upregulated in DENV2-infected cells were linked to terms associated with the cell response, including the response to stress, radiation, and inorganic substances (Figure 2h–i).

### 3.3. ZIKV^PR^ Notably Upregulates Immune Response Genes Compared to ZIKV^MR^ and DENV2

**Figure 3:**
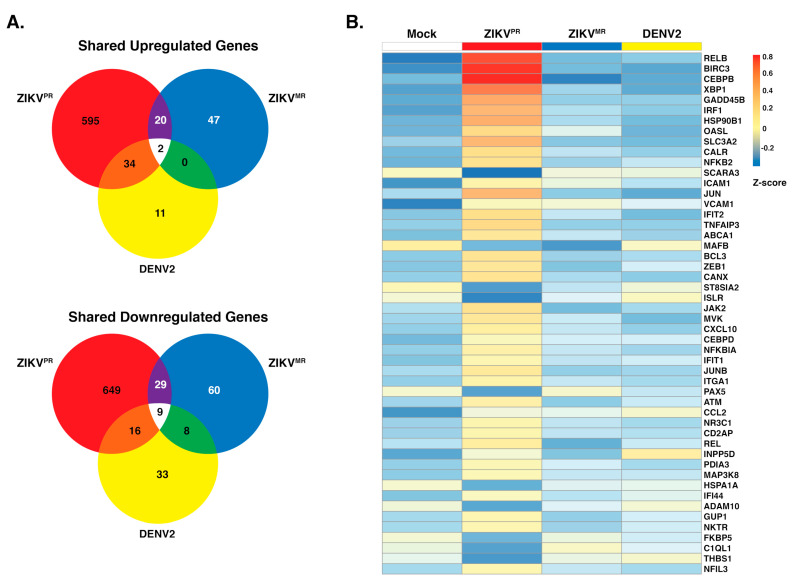
Upregulation and downregulation of genes in SH-SY5Y cells infected with ZIKV^PR^, ZIKV^MR^*,* and DENV2. (**A**) Venn diagram of genes differentially expressed between infected SH-SY5Y cells that were differentially upregulated (top) or downregulated (bottom). DE transcripts from ZIKV^PR^, ZIKV^MR^, and DENV2 are within the respective red, blue, and yellow circles. (**B**) Heatmap of the top 50 statistically significant differentially expressed immune response genes in ZIKV^PR^-infected cells compared to mock. The expression of these genes in ZIKV^MR^ and DENV2-infected SH-SY5Y cells is shown for comparison. Three replicates from each condition were collapsed and normalized to mock. The color scale shows the Z-score of the immune response genes.

Immunofluorescence and viral RNA abundance showed that SH-SY5Y cells were similarly infected by ZIKV^PR^, ZIKV^MR^, and DENV2 (Figure 1). To further understand the biological effect of each virus in SH-SY5Y cells, we used GO analysis to predict effects on cellular processes. We observed that 651 and 703 genes were upregulated and downregulated, respectively, following ZIKV^PR^ infection (Figure 3a). By contrast, only 69 and 106, and 47 and 66 genes were upregulated and downregulated following ZIKV^MR^ and DENV2 infection, respectively (Figure 3a). This differential gene analysis showed only two genes (HECW2 and SATB1) that were upregulated following infection with all three viruses, versus nine common genes (ACHE, PIEZO1, ENO3, CDHR1, CCDC24, VCAM1, SLIT1, C4A, and ZNF321P) that were downregulated (Figure 3a). While 20 upregulated genes were common between the ZIKV isolates and 34 genes were similarly upregulated between ZIKV^PR^ and DENV2, ZIKV^MR^ and DENV2 shared no common genes.

Analysis of the top 25 GO terms in SH-SY5Y cells infected with the ZIKV isolates revealed an effect on immune response genes (Figure 2c). We therefore examined the expression levels of the top 50 statistically significant genes that were categorized as immune response genes between the mock and the three virus infection (Figure 3b). Overall, we noted significant changes in gene expression following ZIKV^PR^ infection compared to mock, ZIKV^MR^, and DENV2 (Figure 3b). Twenty percent of these immune response genes were downregulated following ZIKV^PR^ infection (Figure 3b). RELB, BIRC3, CEBPB, and XBP1 were greatly upregulated in ZIKV^PR^-infected cells (Figure 3b). Innate immune response genes such as IRF1, OASL, IFIT1, and IFIT2, were also all upregulated in ZIKV^PR^-infected cells (Figure 3b and Figure S3).

We observed a handful of genes that were downregulated in both ZIKV-infected cells (Figure 3b). MAFB, ST8SIA2, ISLR, HSPA1A, and ADAM19 are all associated with developmental processes. For example, ADAM19 is known to participate in neuromuscular junction formation [64], while MAFB loss-of-function and dominant-negative mutations result in a congenital eye-movement disorder known as Duane retraction syndrome [65]. Neuromuscular maldevelopment such as arthrogryposis was widely correlated to Zika congenital syndrome [12], and more than 80% of the infants with microcephaly born from ZIKV-infected mothers had ophthalmoscopic abnormalities [12]. Interestingly, ADAM19 and MAFB were both upregulated in DENV-infected cells (Figure 3b).

In ZIKV-infected cells, ICAM1 and VCAM1 were upregulated, but they were downregulated in DENV2-infected cells (Figure 3b). ICAM1 and VCAM1 are involved in immune cell migration across the blood–brain barrier [66,67], where an increase in immune cells in the brain might contribute to the neuropathogenesis associated with intrauterine ZIKV infection. ZIKV and other flaviviruses heavily depend on the secretory pathway for the maturation of viral progeny. It is, therefore, interesting that of the 50 significantly altered genes associated with the immune response in Figure 3b, 21 contained endoplasmic reticulum (ER)-related, Golgi-related, and/or plasma-membrane-related GO cellular component terms within the top five. These findings show that ZIKV^PR^-induced changes in gene expression significantly alter the levels of genes involved in both the immune response and proper cellular development.

When we compared ZIKV^PR^ versus DENV2 DE genes, 138 genes associated with apoptotic signaling were downregulated (Figure S3d). Similarly, 23 apoptotic signaling pathway genes were downregulated when comparing ZIKV^PR^ to ZIKV^MR^, suggesting that the modern isolate of ZIKV has developed strategies to usurp antiviral pathways, allowing for the suppression of the host immune response (Figure S3b). Interestingly, ZIKV^MR^ infection resulted in an upregulation of apoptotic genes when compared to DENV2 (Figure S3e).

### 3.4. Exon Skipping is a Major Splicing Event during ZIKV^PR^, ZIKV^MR^, and DENV2 Infection

**Figure 4:**
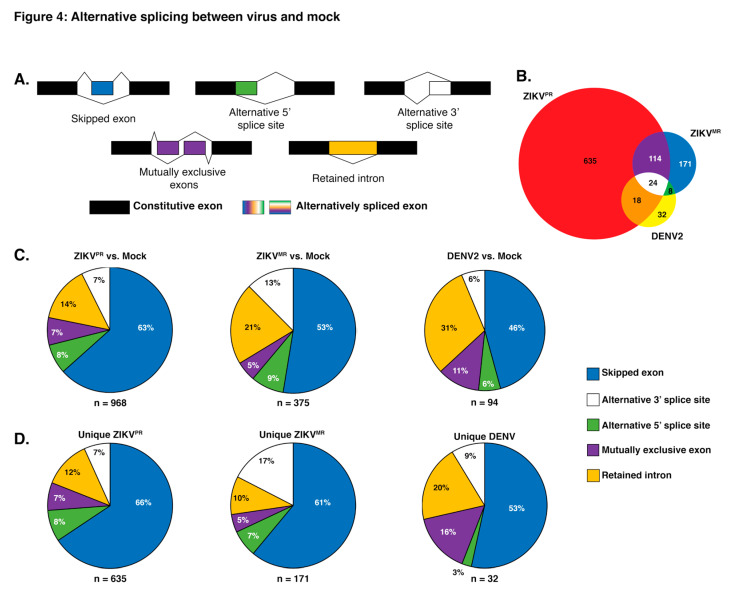
Analysis of alternative splicing events in virus versus mock-infected SH-SY5Y cells. (**A**) Schematic illustrating the five alternative splice events characterized in our analyses. The unchanged exons are black, while the differentially spliced exons are color coded. (**B**) Venn diagram of shared and unique genes that were alternatively spliced between virus and mock-infected cells. Alternative slicing (AS) events from ZIKV^PR^ versus mock, ZIKV^MR^ versus mock, and DENV2 versus mock are highlighted in the red, blue, and yellow circles respectively. (**C**) Pie charts depicting the percentages of all unique AS events for the three viruses versus mock-infected SH-SY5Y cells. (**D**) Pie charts representing the percentages of each type of AS event unique to each virus infection in SH-SY5Y cells. The segment colors match the AS events illustrated in (**A**). Values in (**B**) account for unique genes while (**C**) represents all unique splicing events.

While previous transcriptomic studies have been undertaken during ZIKV infection [22,23,24,25,26], we were interested in changes in alternative splicing (AS) induced following flavivirus infection. The splicing of pre-mRNAs can be separated into five major categories, namely, skipped exons (SE), alternative 5′ or 3’ splice sites, mutually exclusive exons, and the retention of introns (Figure 4a). AS defects resulting in disease can occur when sequences in the pre-mRNA required for correct splicing are mutated or when regulatory factors essential for splicing are mutated [68]. These two scenarios can result in missplicing and thus a reduction in the functional protein product or an imbalanced production of mature mRNA isoforms that contribute to disease.

To analyze splicing events, sequences were aligned to the reference human genome hg19 using STAR with ~93% of reads aligning uniquely in each library. Using rMATs to analyze splice variants, a list of ~77,000 (~55,000 SE events) potential AS events occurring in all replicates was generated. To reduce false-positive splicing events, all AS events with a false discovery rate (FDR) greater than 0.05 and a change in percent spliced in (ΔPSI) of less than 0.1 were omitted (~500–1500 events, depending on libraries). In examining alternative spliced products, we observed that 114 events were shared between both ZIKV isolates and that eighteen AS events were shared between ZIKV^PR^ and DENV2-infected cells (Figure 4b). Similarly to the differential gene expression analysis, infection with the modern Asian-American ZIKV^PR^ isolate resulted in over 2.5 times more AS events compared to cells infected with ZIKV^MR^ or DENV2 (Figure 4c). When analyzing the types of AS event between each virus compared to mock, we observed that skipped exons accounted for 63%, 53%, and 46% of all significant AS events for ZIKV^PR^, ZIKV^MR^, and DENV2, respectively (Figure 4c). The second most prevalent AS event for all infections compared to mock was intron retention (Figure 4c). In DENV2-infected cells, over 30% of AS events were retained introns, while this AS splicing event was lower—at 21% and 14%, respectively—in ZIKV^MR^ and ZIKV^PR^ infected cells (Figure 4b). Interestingly, AS events unique to DENV infection revealed more mutually exclusive exon splicing (16%) compared to the 7% and 5% in ZIKV^PR^ and ZIKV^MR^, respectively (Figure 4c).

**Figure 5:**
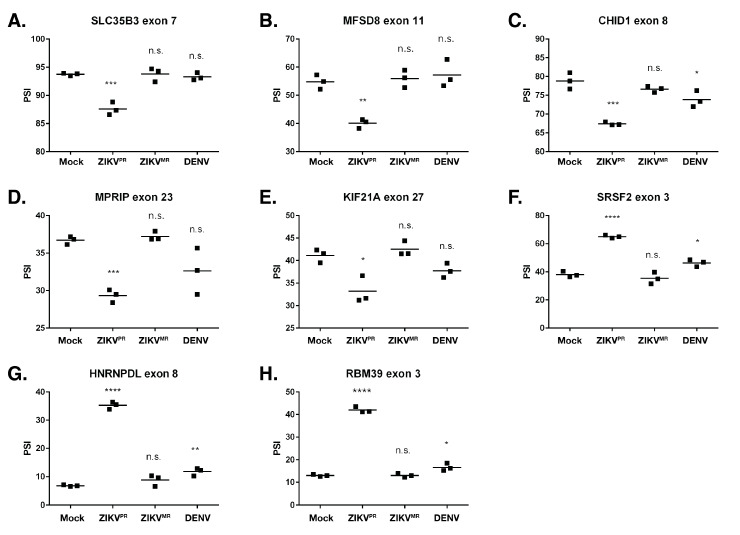
Validation of select alternative splicing events in mock-, ZIKV^PR^-, ZIKV^MR^-, and DENV2-infected SH-SY5Y cells. (**A**–**H**) RNA from each of the three biological replicates was used for RT-PCR for the indicated gene. The percent spliced in (PSI) was calculated as described in the Materials and Methods. PSI indicates the change in the inclusion of a regulated exon. The significance of the data was determined from three independent experiments using the GraphPad software and performing an unpaired Student’s t-test; n.s. denotes not significant, * *p* < 0.05, ** *p* < 0.01, *** *p* < 0.001, and **** *p* < 0.0001. Figure S6 shows the corresponding schematics of the gene exons examined as well as the RT-PCR products and Sashimi plots for the genes analyzed in this figure. Figure S7 shows the correlation between the PSI values obtained from RNA-seq and RT-PCR.

In examining which genes were misspliced in each infection compared to in mock-infected SH-SY5Y cells, we observed 968 genes were alternatively spliced in ZIKV^PR^-infected cells, compared to 375 and 94 for ZIKV^MR^- and DENV2-infected cells, respectively (Figure 4b,c). To further understand the biological processes modulated by AS and virus infection, all AS events for each condition compared to mock-infected cells were analyzed for functional category enrichment (Figure S5a–c). Of the top 10 statistically significant functional categories enriched in cells infected with ZIKV^PR^, four were involved in splicing, providing further support for the observed changes in the global splicing landscape (Figure S5a). A second commonality between the top 10 functional categories was related to the disassembly of various cellular components including organelles, ribonucleoprotein complexes, and ribosomes (Figure S5a). It is possible that the modern Asian-American ZIKV isolate promotes the disassembly of specific cellular factors that would normally limit the viral life cycle. Finally, we observed a significant enrichment in AS genes associated with central nervous system myelination (Figure S5a). When analyzing the AS events during infection with ZIKV^MR^, only eight GO functional categories were statistically significant (Figure S5b). Of these, three were related to protein localization, two of which were involved in nuclear import and export (Figure S5b). Nine significantly enriched categories were found in DENV2-infected cells (Figure 5c). When comparing all significant categories between viral infections, similarities were only found between ZIKV^MR^ and DENV2-infected cells, which included monosaccharide transport, the cellular response to nitrogen compounds, and the regulation of the establishment of protein localization (Figure S5b,c). Moreover, the only significantly enriched functional category strictly specific to the central nervous system was found in cells infected with ZIKV^PR^ (Figure S5a).

We were interested in which genes were uniquely misspliced in each infection. Upon separating all AS events specific to each viral condition, we observed 635 genes were alternatively spliced in ZIKV^PR^-infected cells, compared to 171 and 32 for ZIKV^MR^- and DENV2-infected cells, respectively (Figure 4d). To ascertain whether certain cellular pathways were preferentially being misspliced, all unique AS events in each viral infection were analyzed for enriched GO categories. Each of the top 10 enriched terms for ZIKV^PR^-specific AS events were linked to RNA processing, with six of the ten being associated with splicing terms (Figure S5d). ZIKV^MR^-unique AS events were strongly linked to fatty acid metabolism and apoptotic pathways, while the majority of events resulting from DENV2 infection related to the nervous system (Figure S5e,f).

To validate AS events, we selected 1) transcripts that were misspliced in ZIKV^PR^- compared to ZIKV^MR^- and DENV2-infected cells, and 2) exon inclusion/exclusion events, as these comprised the majority of AS changes and could be easily assessed with RT-PCR. The genes chosen to validate the AS events were selected based on the lowest false discovery rate (FDR) and the highest read count from the RNA-seq data. RT-PCR was used to compare percent spliced in (PSI) values for the chosen events. RT-PCR products were analyzed, and the intensity of the splicing products was quantified (Figure S6). Thus, if virus infection promoted the inclusion of the exon, the PSI value increased. Conversely, if the exon was excluded during virus infection, the PSI value decreased. Moreover, we show Sashimi plots, which illustrate the differences in the splice junctions of the selected transcripts from mock- and ZIKV^PR^-infected SH-SY5Y cells (Figure S6). The genes that were functionally of interest and chosen for validation include: SLC35B3, MFSD8, CHID1, MPRIP, KIF21A, SRSF2, HNRNPDL, and RBM39. HNRNPDL and RBM39 function in transcriptional regulation [69,70], HNRNPDL and SRSF2 regulate alternative splicing [69,71], MPRIP is involved in stress granule formation [62], CHID1 has a role in pathogen sensing [72], and KIF21A has been implicated in neurological diseases [73]. Consistent with the selection criterion, most AS events chosen to be validated via RT-PCR strongly agreed with our RNA-seq data (Figure S7). These data indicate that ZIKV^PR^ infection results in significant missplicing, with the greatest difference in PSI values between mock and ZIKV^PR^ being ~28% (RBM39). For each of the genes examined, we observed no significant splicing changes in the ZIKV^MR^-infected cells when compared to the mock infection. Interestingly, however, in DENV2 infection, we determined small but significantly different changes in exon inclusion/exclusion levels in CHID1, SRSF2, HNRNPDL, and RBM39 (Figure 5).

## 4. Discussion

Here we report on the consequence of ZIKV infection for alternative splicing, an element of cellular RNA homeostasis that is understudied in virus–host interactions. ZIKV has garnered worldwide attention due to the increased incidence of developmental and neurological defects in newborns following intrauterine infection [12,13]. RNA-seq studies in neuronal cells revealed the transcriptional dysregulation of genes associated with cell-cycle, DNA repair, the immune response, cell death, and microcephaly [23,24,27,28,29,35]. To investigate alternative splicing in neuronal cells, we sought a human cell culture line that would model ZIKV infection in neurons. To this end we used the neuroblastoma cell line SH-SY5Y, which has previously been used to investigate virus–host interactions with different neurotropic viruses such as poliovirus [74], enterovirus D68 [75], herpes viruses [76,77,78], and Japanese encephalitis virus [79]. We observed that the infection of SH-SY5Y cells with the two different ZIKV strains and DENV2 for 24 h at a moi of 5 resulted in similar levels of viral RNA and infectious viral particles (Figure 1c,d). Initial studies examining gene dysregulation following ZIKV infection were undertaken in human neural stem cells (hNSCs) [30,31], human neural progenitor cells (hNPs) [27,32], organotypic cultures [29,33], neurospheres [34], and cerebral organoids [35]. These studies used different ZIKV strains, multiplicities of infection, and lengths of infection, and thus the extent of ZIKV infection ranged from 2.5% to 90% [27,29,30,31,32,33,34,35]. Consistent with earlier reports of ZIKV infection in SH-SY5Y cells [80,81], immunofluorescence analyses revealed that 40% of cells were infected (Figure 1a,b). While we recognized that the differential gene expression and alternative splicing analyses collected would be on a modest number of infected cells, we reasoned that this level of infection would be comparable to the previous transcriptome-wide studies [27,29,30,31,32,33,34,35] and that, thus, changes in the alternative splicing landscape would model such changes in ZIKV-infected neurons.

Our RNA-seq studies demonstrated that in SH-SY5Y cells, ZIKV^PR^ alters the transcriptional landscape more than either ZIKV^MR^ or DENV2 (Figure 2b). GO term analyses of the differentially expressed genes showed that during ZIKV but not DENV2 infection, innate immune response genes were modulated (Figure 2c). In primary human skin fibroblasts, the French Polynesia ZIKV isolate (ZIKV^FP^) promoted the transcription of *RIG-I* (*DDX58*), *MDA5* (*IFIH1*), and *TLR3*, as well as interferon-stimulated genes such as *OAS2*, *ISG15*, and *MX1* [82]. We similarly found that *RIG-I* (*DDX58*) and *MDA5* (*IFIH1*) were upregulated by both ZIKV isolates, but not by DENV2 (Figure S4). The 2′,5′-oligoadenylate synthase (OAS) and RNase L pathways have previously been shown to restrict flavivirus infection [83,84,85], yet in ZIKV^PR^-infected SH-SY5Y cells, we observed that *OAS3* was downregulated (Figure S4), while *OASL* was notably upregulated (Figure 3b). OASL was previously shown to have antiviral activity mediated through RIG-I activation [73]. It is therefore possible that in neuronal cells, ZIKV gene expression is restricted by a RIG-I-OASL-linked and/or MDA5 (IFIH1) pathway. We also found that NF-kB inflammatory response genes including *BIRC3*, *NFKBIA*, *REL*, and *CXCL10* were upregulated in ZIKV^PR^-infected cells (Figure 3). These genes were similarly upregulated in ZIKV-infected human neural progenitor cells [86] and primary human skin fibroblasts infected with ZIKV^FP^ [82], although Simonin et al., reported that in human primary neuronal cells infected with an Asian ZIKV strain, NF-kB genes were downregulated [30]. Overall, our RNA-seq analyses of ZIKV-infected SH-SY5Y cells show similar transcriptional changes in cellular processes to studies in organoids and neural stem and progenitor cells. The differences between studies of differentially expressed genes might in part be due to the infection system, variability in infection levels, and ZIKV isolates used.

We posited that in addition to altering gene expression, ZIKV infection would also impact alternative splicing. Notably, herpes viruses, adenovirus, and influenza A virus subvert the cellular RNA splicing machinery to broaden the coding capabilities of the viral genome [87,88,89], and Sindbis virus has been shown to alter the polyadenylation and splicing of select cellular mRNAs by sequestering cellular RNA binding proteins involved in these processes [38]. Although flaviviruses do not splice their own RNA genomes, DENV2 RNA-dependent RNA polymerase (NS5) has been shown to localize in the nucleus and bind to and interfere with the mRNA splicing of multiple antiviral factors [90]. Here, we identified 968 and 375 AS events in ZIKV^PR^- and ZIKV^MR^-infected cells, respectively (Figure 4b). The skipping of an exon cassette is the most common type of AS in humans [91,92]. Indeed, following infection with all three viruses, our analyses revealed that exon skipping accounted for the majority (45–63%) of all AS events (Figure 4a–c). Thirteen percent of splicing reactions used an alternative 3′ splice site in ZIKV^MR^-infected cells, compared to 7% and 6% in ZIKV^PR^- and DENV2-infected SH-SY5Y cells, respectively (Figure 4c). In reovirus-infected cells, alternative 3′ splice site event selection occurred in 24% [93]. Of the different types of AS we analyzed, 14% of the AS events in ZIKV^PR^-infected cells were intron-retention. In reovirus-infected cells, intron retention accounted for just 10% of AS types [93]. While intron retention was initially thought to be the least prevalent form of AS in animals, Braunschweig et al. (2014) reported that intron retention was a frequent occurrence and that this mode of AS functioned to broadly reduce the levels of transcripts that were not required for cellular homeostasis [94]. In ZIKV^MR^- and DENV2-infected cells, 21% and 31% of AS events were via intron retention (Figure 4c). It would be interesting to investigate the turnover rate of the intron-retained transcripts in ZIKV^MR^- and DENV2-infected cells and compare these with the intron-retained transcripts in ZIKV^PR^-infection. One intriguing possibility is that the change in RNA stability might limit the effect of ZIKV^MR^ and DENV2 on neuropathogenesis.

Since ZIKV^PR^ infection resulted in the highest number of AS transcripts and that many of these were categorized within mRNA splicing-related processes (Figure S5), we focused our validation on AS events arising during ZIKV^PR^ infection. We specifically validated *SLC35B6*, *MFSD8, CHID1, MPRIP, KIF21A, SRSF2, HNRNPDL,* and *RBM35*. These genes were of particular interest as alternative splicing could influence ZIKV infection and/or neuron development or function. For example, the alternative splicing of *SLC35B6*, *MFSD8*, and *CHID1* might affect ZIKV infection. ZIKV enters the cells by receptor-mediated endocytosis, where membrane fusion of the viral envelope is triggered by low pH and the glycosylated envelope proteins [1]. SLC35B3 localizes primarily to the ER and Golgi and is a 3′-phophoadenosine 5′-phosphosulfate transporter involved in sulfation [95], and MFS domain containing 8 (*MFSD8*), also known as *CLN7*, encodes a ubiquitously expressed putative lysosomal transporter [96]. Thus, the alternative splicing of *SLC35B6* and *MFSD8* might alter functions within the endomembrane system and affect ZIKV entry and/or egress. Interestingly, mutations in *CLN7* have been linked to a group of autosomal recessive neurodegenerative diseases known as neuronal ceroid lipofuscinoses (NCLs) [97]. Last, Chitinase Containing Domain protein 1 (*CHID1)* encodes a protein belonging to the superfamily glycoside hydrolase family 18 (GH18) and may have a role in carbohydrate binding [72]. CHID1 has been shown to bind lipopolysaccharide (LPS), suggesting this protein plays roles in pathogen sensing [72]. It is possible that the exon exclusion event occurring in ZIKV^PR^-infected cells (Figure 5c) specifically targets a domain required for pathogen sensing to promote or limit infection.

Myosin phosphatase-Rho interacting protein (MPRIP) targets myosin phosphatase to the actin cytoskeleton and is required for the regulation of actin by RhoA and ROCK1 [98]. Curiously, MPRIP overexpression has been shown to disassemble stress granules (SGs) in neuronal cells [99]. In addition to the increased exclusion of exon 23 (Figure 5d), our transcriptomic studies revealed an increased expression of *MPRIP* only in ZIKV^PR^-infected cells. ZIKV disrupts the formation of sodium arsenite-induced SGs in neuronal and non-neuronal cells [36,100], and we have shown that specific SG components exhibit both proviral and antiviral functions [36]. While the exact composition of neuronal SGs is not known, perhaps different *MPRIP* isoforms differentially modulate the formation of neuronal SGs, which would impact ZIKV gene expression. We also showed that ZIKV^PR^ infection led to the increased exclusion of exon 27 in *KIF21A* (Figure 5e). KIF21A is a member of the KIF4 subfamily of kinesin-like motor proteins that has been shown to affect axon and growth cone morphology [73], presenting the possibility that the alternative splicing of *KIF21A* as a result of ZIKV infection influences neurodevelopment.

SRSF proteins constitute a large portion of the spliceosome, contain RNA recognition motifs (RRMs), and also play a role in the export of mRNA from the nucleus [101]. Our analyses showed that *SRSF2*, *SRSF3*, *SRSF6*, and *SRSF7* were alternatively spliced in ZIKV^PR^-infected cells but not in ZIKV^MR^-infected cells. HNRNPDL (JKTBP1) is a paralog of HNRNPD (AUF1), which has been shown to be important for transcriptional regulation and alternative splicing via binding to AU-rich sequences [69]. Hu et al. similarly reported that *HNRNPDL* was alternatively spliced in ZIKV^PR^-infected cortical neural progenitor cells [102]. The inclusion of exon 8 targets *HNRNPDL* for nonsense-mediated decay (NMD) [103], presenting the possibility that by promoting exon 8 inclusion (Figure 5g), ZIKV^PR^ infection targets *HNRNPDL* for degradation. Lastly, the downregulation of the splicing factor RNA binding motif protein 39 (*RBM39*) decreases the expression of cell-cycle progression regulators. Since ZIKV has been shown to affect the cell cycle [23,27], it would be interesting to determine how the alternative splicing of *RBM39* contributes to this process (Figure 5h). As it modulates the exon incorporation/skipping of *SRSF2*, *HNRNPDL*, and *RBM39* alone, it is not surprising that ZIKV^PR^ changed the global alternative splicing landscape in infected cells (Figure 5f–h, and Figure S5f–h), a change that could significantly impact the infectious cycle and pathogenesis.

Gene levels change dramatically during brain development. Such changes are not just directed by transcript levels but also by alternative splicing, which can expand the proteome. Moreover, alternative splicing may influence RNA stability, the efficiency of translation, and localization. It is therefore hardly surprising that alternative splicing is intimately involved in neurodevelopment and synaptic plasticity. Devastating congenital abnormalities and neurological complications were linked to infection during the recent ZIKV outbreak [12,13], and transcriptome-wide studies have provided initial insight into the molecular neuropathogenesis of ZIKV [14,22,23,24,25,27,28,29]. Here, we demonstrate that changes in gene expression are not the only effect ZIKV has on cellular RNA homeostasis, but that ZIKV also modulates alternative splicing events. By identifying and validating AS events resulting from ZIKV^PR^, we have established the foundation for future studies to dissect the biological impact of ZIKV-induced changes to the alternative splicing landscape and neurodevelopment.

## Supplementary Materials

The following are available online at https://www.mdpi.com/1999-4915/12/5/510/s1, Table S1: List of primers used for RT-qPCR and validation of alternatively spliced mRNAs, Figure S1: Volcano plots of differential gene expression analysis, Figure S2: Analysis of the top 10 differentially expressed genes, Figure S3: Analysis of GO terms of upregulated and downregulated genes in virus-infected SH-SY5Y cells, Figure S4: Heatmap of differentially expressed interferon-related genes in mock- and virus infected SH-SY5Y cells, Figure S5: GO term analysis of alternatively spliced events in mock- and virus infected SH-SY5Y cells, Figure S6: Schematic, gel analysis of RT-PCR products and Sashimi plots of eight validated alternatively spliced targets, and Figure S7: Correlation between RNA-seq- and RT-qPCR-derived PSI values.
