# Supplementary material for: Asian Zika Virus Isolate Significantly Changes the Transcriptional Profile and Alternative RNA Splicing Events in a Neuroblastoma Cell Line
Source: Viruses. 2020 May 5;12(5):510. doi: 10.3390/v12050510 (PMC7290316; doi:10.3390/v12050510)
Supplement: Supplementary file 1 [file viruses-12-00510-s001.pdf]

Asian Zika virus isolate significantly changes the transcriptional profile and alternative RNA splicing events in a neuroblastoma cell line

Gaston Bonenfant, Ryan Meng, Carl Shotwell, Pheonah Badu, Anne F. Payne, Alexander T. Ciota, Morgan A. Sammons, J. Andrew Berglund, and Cara T. Pager.

Supplemental Data:

Table S1.	List of primers used for RT-qPCR and validation of alternatively spliced mRNAs.
Figure S1.	Volcano plots of differential gene expression analysis.
Figure S2.	Analysis of the top 10 differentially expressed genes.
Figure S3.	Analysis of GO terms of upregulated and downregulated genes in virus-infected SH-SY5Y cells.
Figure S4.	Heatmap of differentially expressed interferon-related genes in mock- and virus infected SH-SY5Y cells.
Figure S5.	GO term analysis of alternatively spliced events in mock- and virus infected SH-SY5Y cells.
Figure S6.	Schematic, gel analysis of RT-PCR products and Sashimi plots of eight validated alternatively spliced targets.
Figure S7.	Correlation between RNA-seq- and RT-qPCR-derived PSI values.

Table S1: List of primers used for RT-qPCR and validation of alternatively spliced mRNAs

Primer target	Sequence (5’ → 3’)
$ZIKV^{MR}$ (FWD)	TTGGTCATGATACTGCTGATTGC
$ZIKV^{MR}$ (REV)	CAGGTCCCACCTGACATGC
$ZIKV^{PR}$ (FWD)	CCTTGGATTCTTGAACGAGGA
$ZIKV^{PR}$ (REV)	AGAGCTTCATTCTCCAGATCAA
DENV2 (FWD)	CAGGTTATGGCACTGTCACGAT
DENV2 (REV)	CCATCTGCAGCAACACCATCTC
β-Actin (FWD)	GTCACCGGAGTCCATCACG
β-Actin (REV)	GACCCAGATCATGTTTGAGACC
HNRNPDL (FWD)	CAACAGAGCACTTATGGCAAGG
HNRNPDL (REV)	CGTCCTGCAAGATGGGTTACT
SRSF2 (FWD)	AGGAGCGGTGTCCTCTTAAGA
SRSF2 (REV)	TTTTTCCCCAAGTCCTCCGTT
RBM39 (FWD)	CCGAACACGAGCACCACAG
RBM39 (REV)	GTTCTTCATGGCCGTTGGCA
MPRIP (FWD)	GATCCTGTGTCACCCGGCAA
MPRIP (REV)	CCGTTCTTGCACCGTCAG
KIF21A (FWD)	CCAGGCAGTCATCTCTATCAGA
KIF21A (REV)	GCACAGCTTTTGTATGCCCT
CHID1 (FWD)	GCTTCGTGGTGGAGGTCTG
CHID1 (REV)	GGGCCAGCTGCTCAAACTC
MFSD8 (FWD)	TACTTGCTGCTCTTGGGGT
MFSD8 (REV)	TTCCCCAAATGTGGTATTAGGG
SLC35B3 (FWD)	TGACAGCACAACTGCACCA
SLC35B3 (REV)	GCCTAATCCACTAGTGCATGT

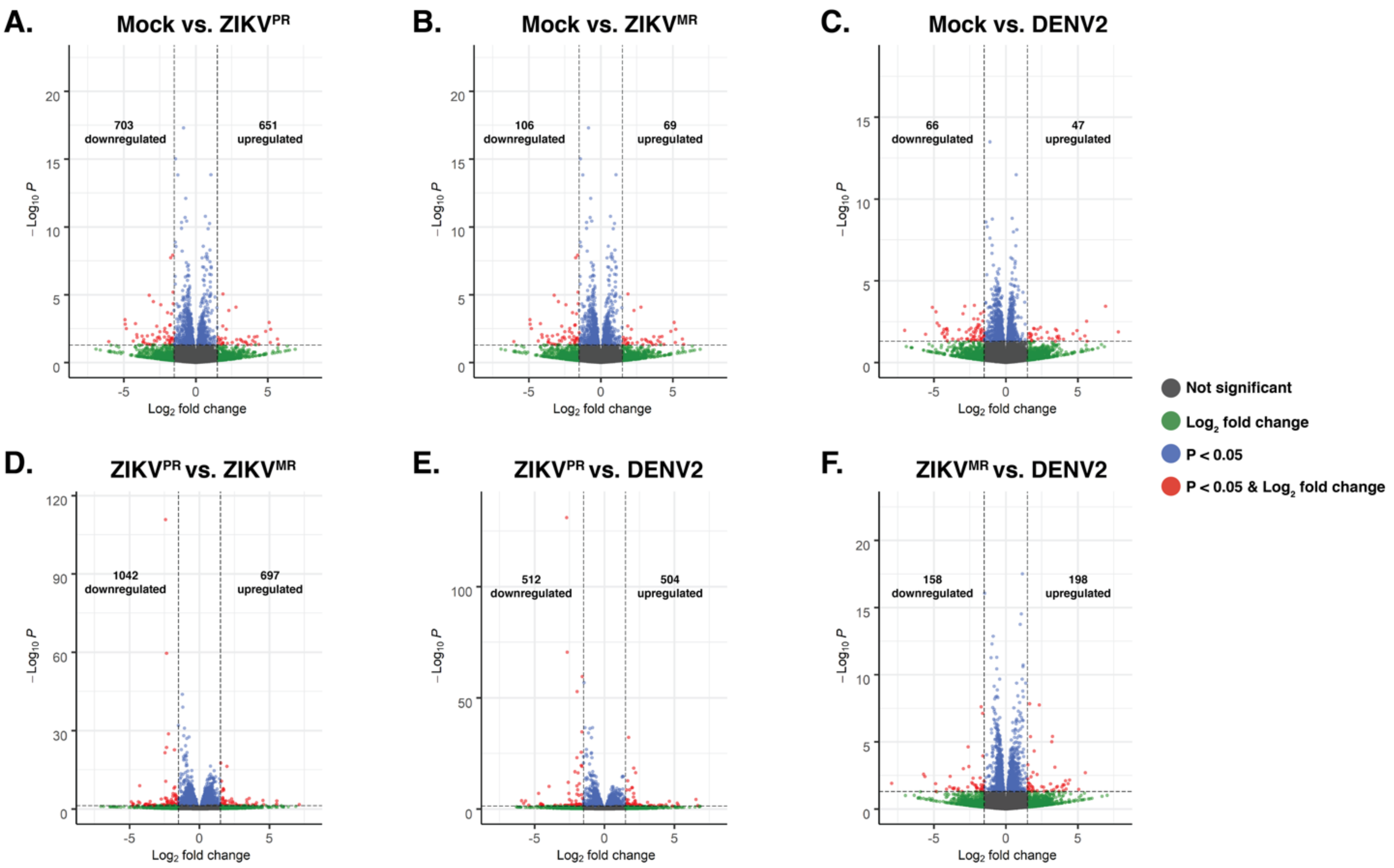

Figure S1: Volcano plots of differential gene expression analysis in mock- and virus-infected SH-SY5Y cells.
A–F) Volcano plots of differential gene expression analysis plotting the -$Log_{10}P$ versus Log_2 fold change for the indicated comparison. Values overlaid on each graph represent number of downregulated (left) and upregulated (right) genes for the indicated condition.

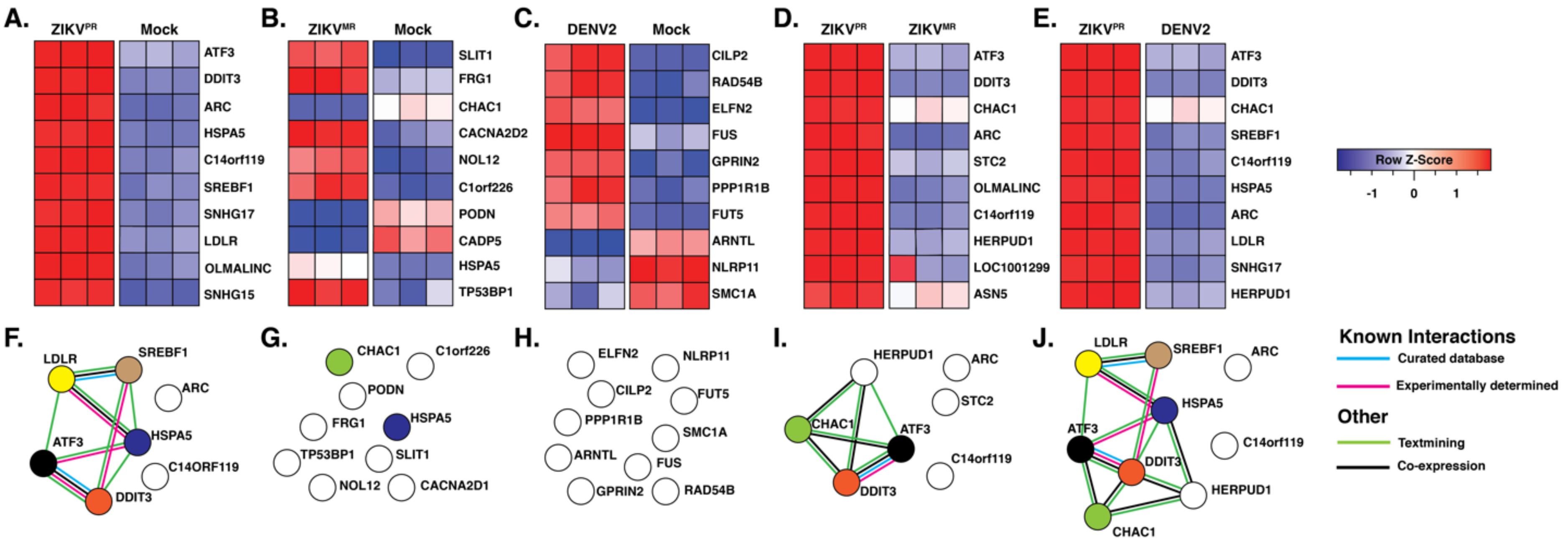

$ZIKV^{PR}$ vs. Mock	$ZIKV^{MR}$ vs. Mock	DENV2 vs. Mock	$ZIKV^{PR}$ vs. $ZIKV^{MR}$	$ZIKV^{PR}$ vs. DENV2	
7	9	10	7	9	Number of nodes
8	0	0	6	14	Number of edges
2.29	0	0	1.71	3.11	Average node degree
2.07E-07	1	1	1.37E-09	2.49E-14	PPI enrichment p-value

Figure S2: Analysis of the top 10 differentially expressed genes.
A-E) Heatmaps generated using Limma-voom. Shaded squares represent the number of standard deviations away from the mean (Z-score) for each of three biological replicates. F-J) Known (blue and pink lines) and predicted (green and black lines) interactions between the top 10 differentially expressed genes of each comparison. Listed below node maps are the statistics generated from STRING.

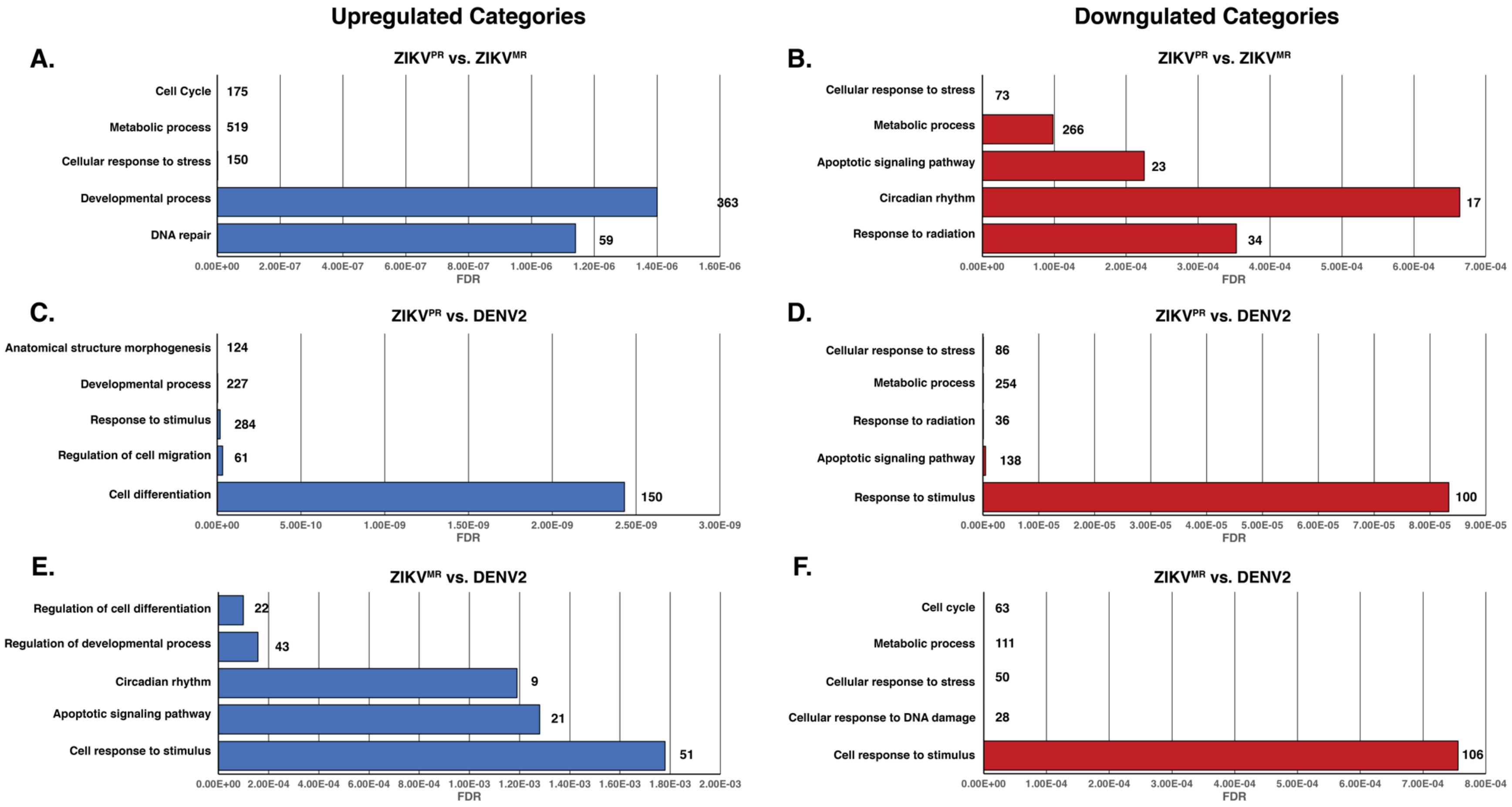

Figure S3: Analysis of GO terms of upregulated and downregulated genes in virus-infected SH-SY5Y cells.
All statistically significant genes that were differentially upregulated (blue) or downregulated (red) were input into enrichR. The top 5 categories were plotted. Numbers to right of bars indicate number of genes in particular category. GO terms are shown on the Y-axes, and false discovery rates (FDR) are represented on the X-axes.

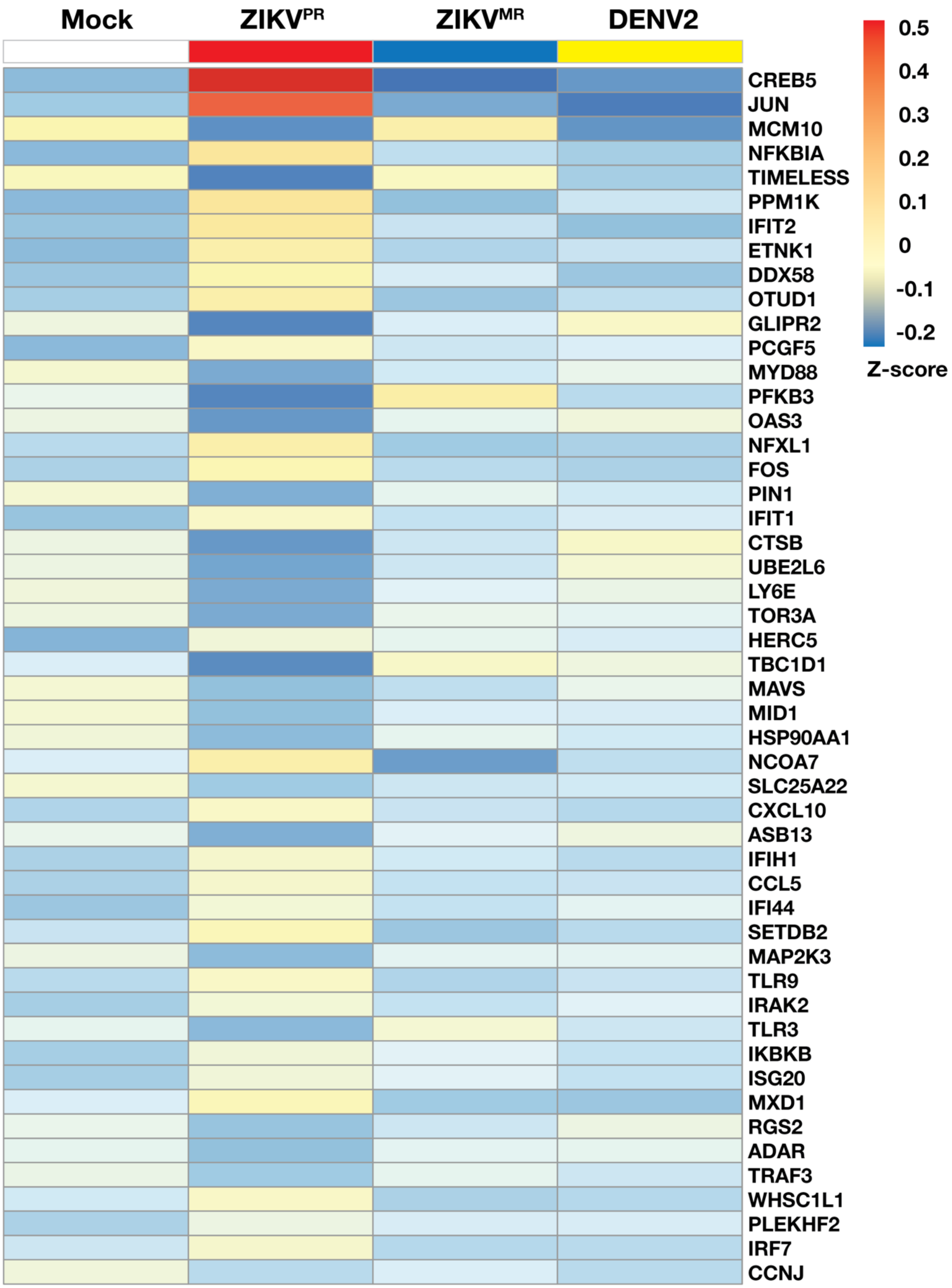

Figure S4: Heatmap of differentially expressed interferon-related genes in mock- and virus infected SH-SY5Y cells. Heatmap of the top 50 differentially expressed genes related to the interferon pathways in ZIKV^{PR}-infected cells compared to mock. Three replicates from each condition were collapsed and compared to mock. The color scale shows the Z-score of

the immune response genes. The change in levels between Mock and ZIKV^{PR} was significant ($p < 0.05$) for *CREB5, JUN, NFKBIA, PPM1K, IFIT2, DDX58, OTUD1, GLIPR2, NFXL1, FOS, IFIT1, TLR9, MXD1* and *IRF7*.

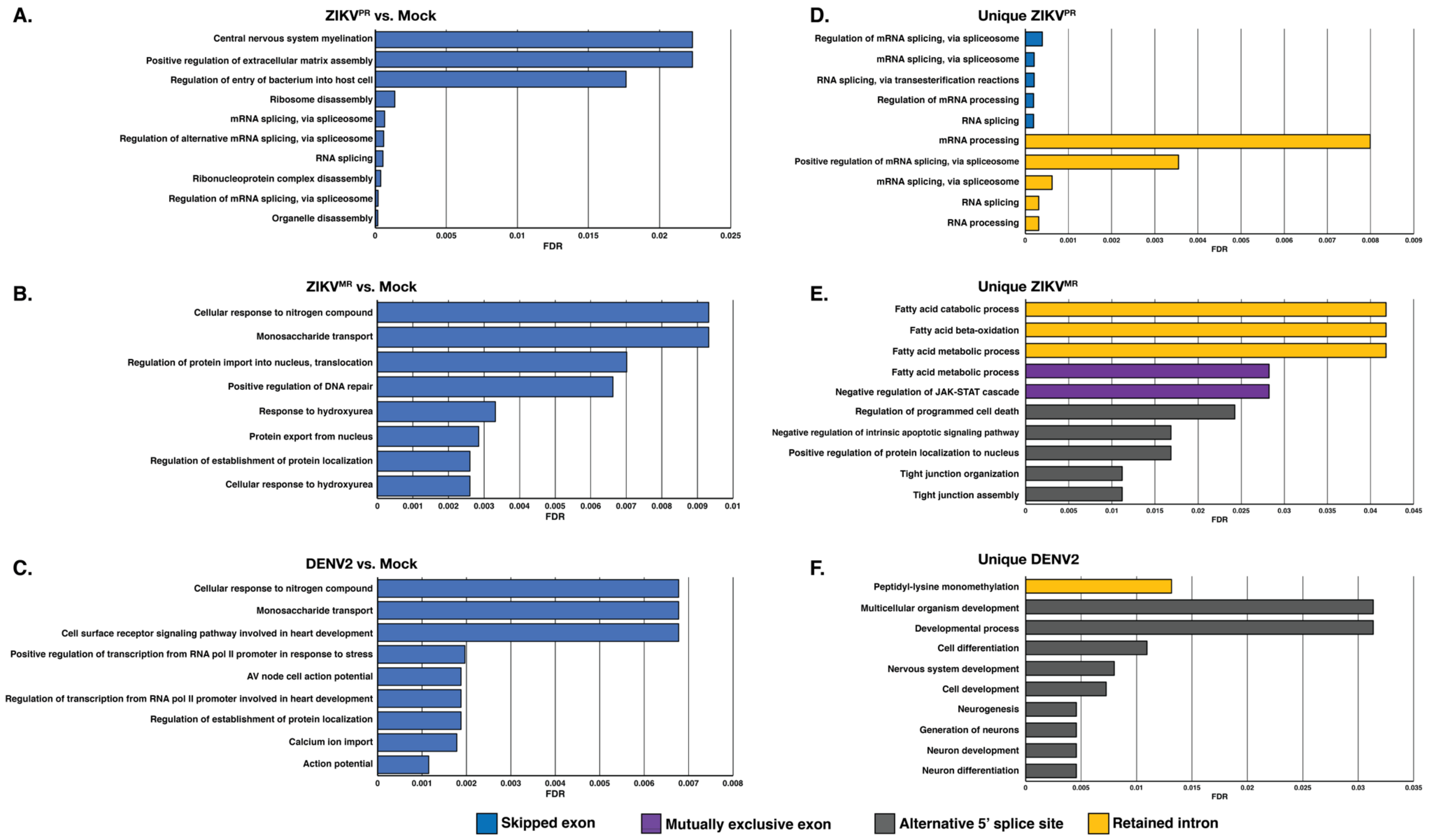

Figure S5. GO term analysis of alternatively spliced events in mock- and virus infected SH-SY5Y cells.

All gene IDs of a given subset were input into ShinyGO(2.0) for functional enrichment. The top 10 functionally enriched categories were plotted. A, B, & C) Analyses of the cellular processes enriched when comparing virus versus mock-infected SH-SY5Y cells. D, E, & F) GO categorization of virus-specific alternatively spliced events. GO terms are annotated on the Y-axes, and false discovery rates (FDR) are represented on the X-axes.

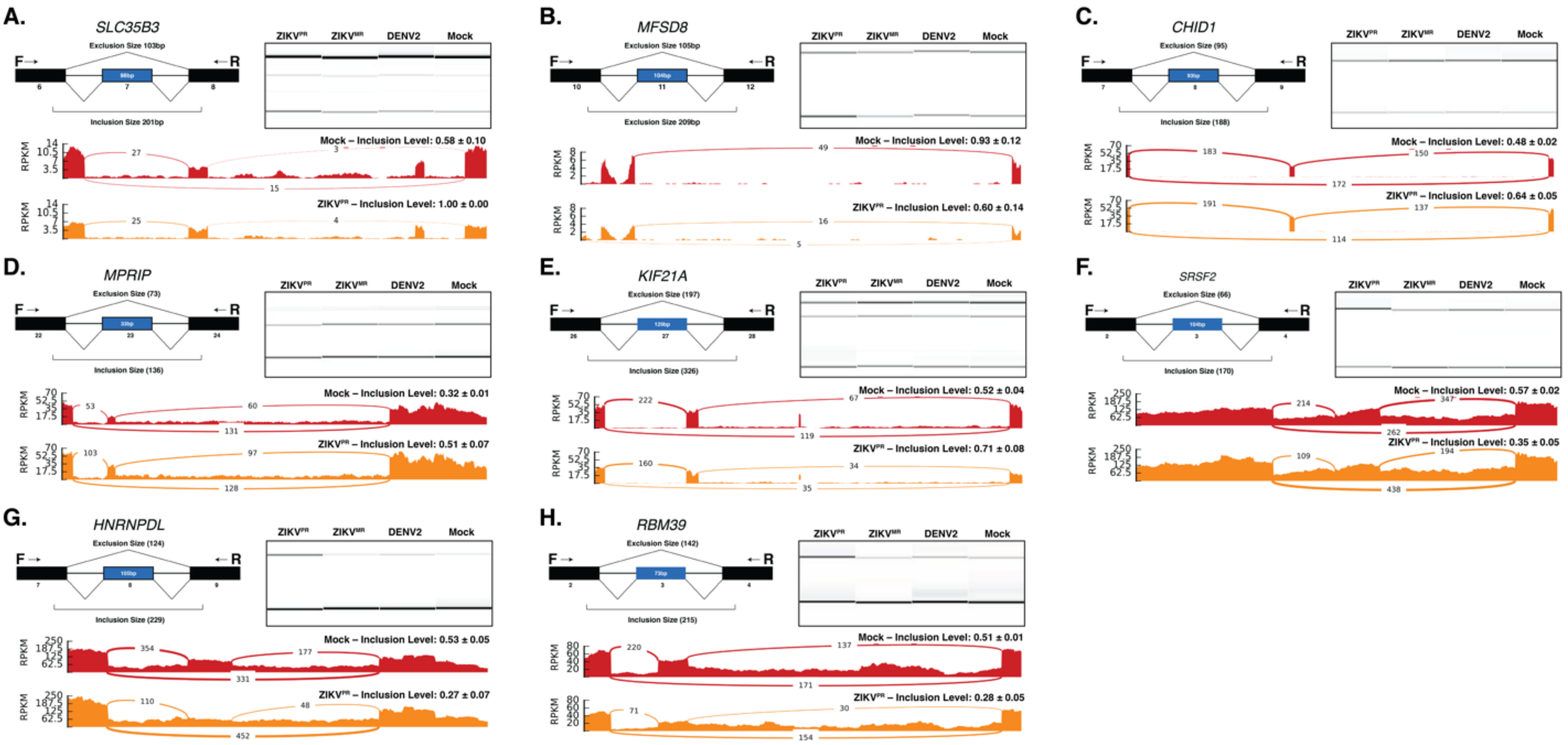

Figure S6: Schematic, gel analysis of RT-PCR products and Sashimi plots of eight validated alternatively spliced targets.
For each panel: (top left) a schematic illustrating the constitutive exons (black) and the cassette exon (blue) of interest. Predicted exclusion and inclusion sizes based off primer binding sites are shown; (top right) representative gel image of alternative splicing analysis where top band represents inclusion of the cassette exon and the lower band represents exclusion of the cassette exon; (bottom) one representative Sashimi plot from Mock-infected (red) and $ZIKV^{PR}$ infected (orange) SH-SY5Y cells, from each transcript validated. Values listed for inclusion levels represent the average of all three biological replicates ± standard deviation.

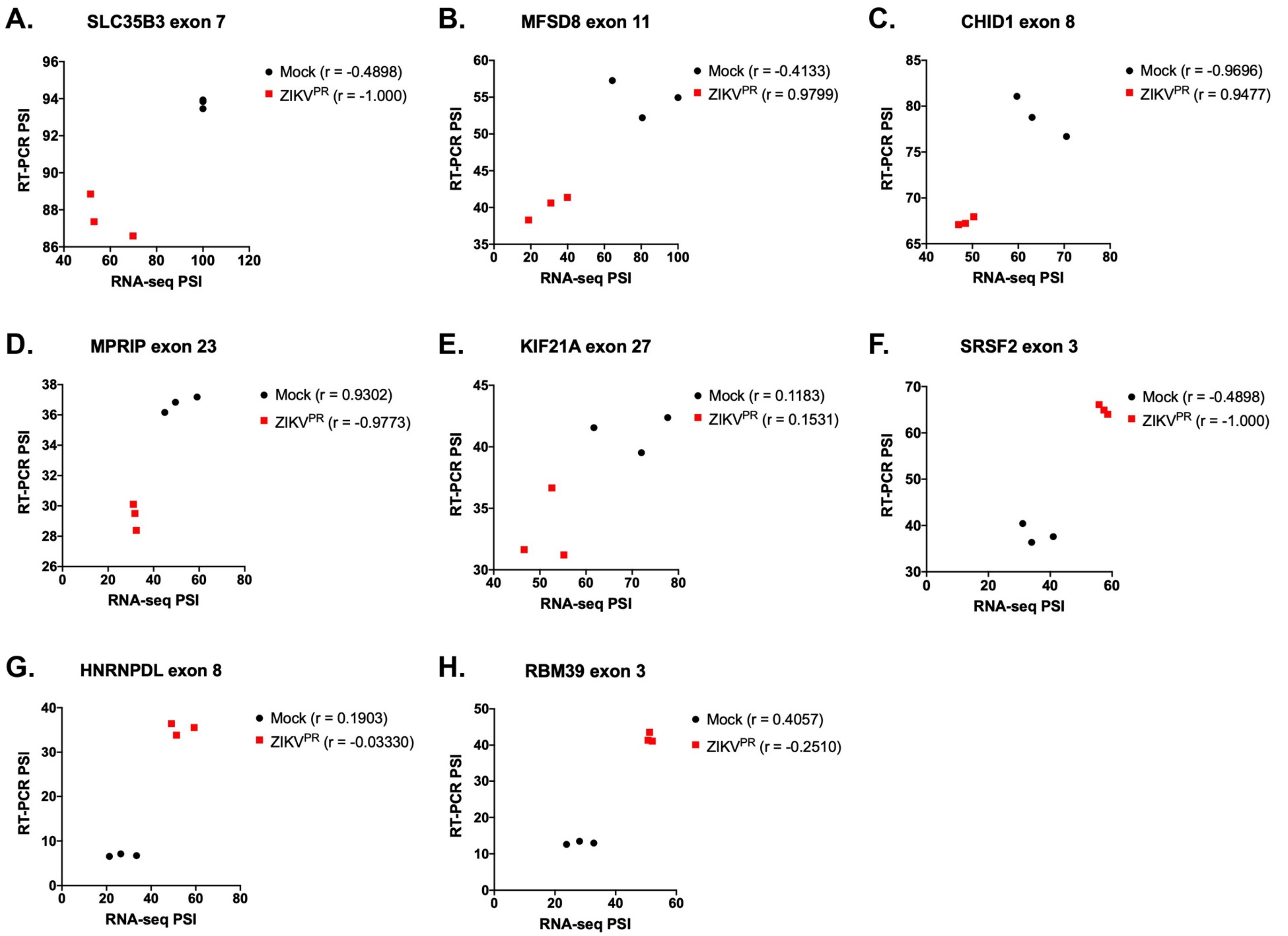

Figure S7: Correlation between RNA-seq- and RT-PCR-derived PSI values.

Percent spliced in (PSI) values from three independent experiments for the indicated genes determined from RNA-seq data were plotted against RT-PCR generated PSI values to determine the correlation. Pearson's correlation coefficient (R) are reported for mock- and ZIKV^{PR}-infected SH-SY5Y cells.
